# 

**Published:** 1886-12

**Authors:** 


### CONTENTS.

PROCEEDINGS .......... 583

COMMUNICATIONS.

Remarks on the Prevention of Scrivener's Palsy or Writer's Cramp.... 610

SELECTIONS.

Oral Disease and Caseous Phthisis.......... 616
Esmarch's Bandage in Anæsthesia.......... 619
A Fungus Developed in the Human Saliva.......... 620
Thinking and Working.......... 620
Minutes of the Ohio State Dental Society.......... 621
SALMAGUNDI .......... 626

EDITORIAL.

Obituary.......... 630

